# Correction: Genchi et al. Understanding the Role of Alcohol in Metabolic Dysfunction and Male Infertility. *Metabolites* 2024, *14*, 626

**DOI:** 10.3390/metabo16070484

**Published:** 2026-07-10

**Authors:** Valentina Annamaria Genchi, Angelo Cignarelli, Andrea Sansone, Dimitri Yannas, Leonardo Dalla Valentina, Daniele Renda Livraghi, Giorgia Spaggiari, Daniele Santi

**Affiliations:** 1 Department of Precision and Regenerative Medicine and Ionian Area-Section of Internal Medicine, Endocrinology, Andrology and Metabolic Diseases, University of Bari Aldo Moro, 70121 Bari, Italy; 2 Chair of Endocrinology and Medical Sexology (ENDOSEX), Department of Systems Medicine, University of Rome Tor Vergata, Tower E South, Room E 413, Via Montpellier 1, 00133 Rome, Italy; 3 Department of Biomedical, Metabolic and Neural Sciences, University of Modena and Reggio Emilia, 41121 Modena, Italydaniele.santi@unimore.it (D.S.); 4 Unit of Endocrinology, Department of Medical Specialties, Azienda Ospedaliero-Universitaria of Modena, 41125 Modena, Italy

The authors would like to make the following correction to their published paper [1].

The change is as follows:

Reference [88] in the original publication is incorrectly cited and needs to be replaced with the following reference:

88. Slevin, E.; Baiocchi, L.; Wu, N.; Ekser, B.; Sato, K.; Lin, E.; Ceci, L.; Chen, L.; Lorenzo, S.R.; Xu, W.; et al. Kupffer Cells: Inflammation Pathways and Cell-Cell Interactions in Alcohol-Associated Liver Disease. *Am. J. Pathol.* **2020**, *190*, 2185–2193.

The authors state that the scientific conclusions are unaffected. This correction was approved by the Academic Editor. The original publication has also been updated.
